# Identification and heterologous expression of the globomycin biosynthetic gene cluster

**DOI:** 10.1016/j.synbio.2023.02.001

**Published:** 2023-02-07

**Authors:** Daniel Oves-Costales, Tetiana Gren, Eva Baggesgaard Sterndorff, Jesús Martín, Francisco Javier Ortiz-López, Tue S. Jørgensen, Xinglin Jiang, Fernando Román-Hurtado, Fernando Reyes, Olga Genilloud, Tilmann Weber

**Affiliations:** a Fundacion MEDINA, Centro de Excelencia en Investigación de Medicamentos Innovadores en Andalucía, Avda del Conocimiento 34, 18016, Armilla, Granada, Spain; b The Novo Nordisk Foundation Center for Biosustainability, Technical University of Denmark, Kemitorvet, building 220, 2800 Kgs. Lyngby, Denmark

**Keywords:** Globomycin, Signal peptidase II, Cyclic lipodepsipeptide, Genome mining, CRISPR-cBEST, Heterologous expression

## Abstract

Globomycin is a cyclic lipodepsipeptide originally isolated from several *Streptomyces* species which displays strong and selective antibacterial activity against Gram-negative pathogens. Its mode of action is based on the competitive inhibition of the lipoprotein signal peptidase II (LspA), which is absent in eukaryotes and considered an attractive target for the development of new antibiotics. Despite its interesting biological properties, the gene cluster encoding its biosynthesis has not yet been identified. In this study we employed a genome-mining approach in the globomycin-producing *Streptomyces* sp. CA-278952 to identify a candidate gene cluster responsible for its biosynthesis. A null mutant was constructed using CRISPR base editing where production was abolished, strongly suggesting its involvement in the biosynthesis. The putative gene cluster was then cloned and heterologously expressed in *Streptomyces albus* J1074 and *Streptomyces coelicolor* M1146, therefore unambiguously linking globomycin and its biosynthetic gene cluster. Our work paves the way for the biosynthesis of new globomycin derivatives with improved pharmacological properties.

## Graphical abstract

**Figure:**
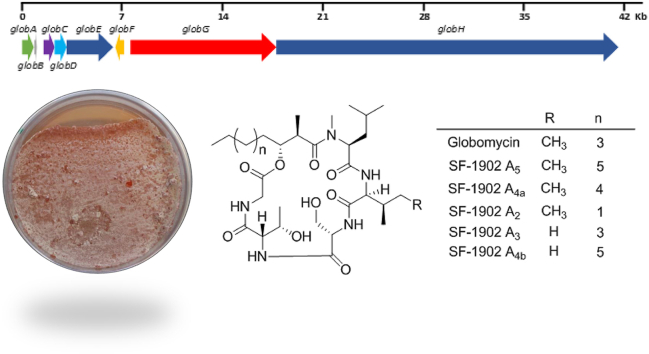

## 1 Introduction

Antimicrobial resistance (AMR) coupled with dwindling antimicrobial drug pipelines poses enormous challenges for the treatment of infectious diseases [1]. It has recently been estimated that in 2019 AMR was directly or indirectly responsible for an estimated 4.95 million deaths globally [2]. The situation is particularly critical with Gram-negative pathogens, with *Escherichia coli*, *Klebsiella pneumoniae*, *Acinetobacter baumannii* and *Pseudomonas aeruginosa* among the six top pathogens causing global deaths. Indeed, these four pathogens were identified as “Priority 1: critical” by the WHO in their priority pathogens list for R&D of new antibiotics [3]. Therefore, new antibiotics, ideally with new mode of actions, are urgently needed.

The lipoprotein maturation pathway, responsible of the maturation and translocation of lipoproteins from the cytosol to the outer membrane, is considered to be essential for many Gram-negative pathogens. It has no equivalent in eukaryotes and therefore has been proposed as an attractive target for the development of new antimicrobials [[4], [5], [6]]. Three proteins from the early steps of this pathway, Lgt (diacylglyceryl transferase), LspA (type II signal peptidase) and Lnt (*N*-acyltransferase) have been functionally and structurally characterized, standing out as the most attractive targets. Currently, only two natural products are known to inhibit the function of the LspA signal peptidase: myxovirescin, which is produced by myxobacteria [7], and globomycin.

**Fig. 1:**
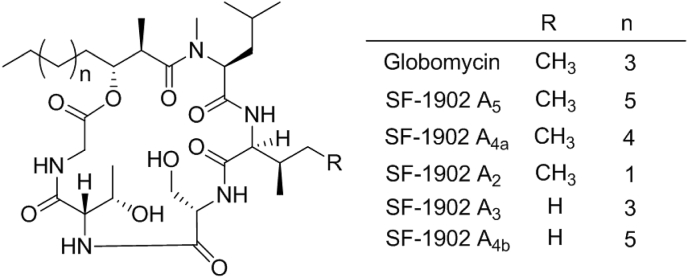
Chemical structures of globomycin and related congeners.

Globomycin is the name given to the most abundant component of a family of cyclic lipodepsipeptides originally isolated from several *Streptomyces* strains [[8], [9], [10]], which shows potent activity against Gram-negative bacteria. Its structure comprises an alkyl chain of variable length attached to a cyclic pentapeptide core which contains the non-proteinogenic amino acids L-*allo*-threonine in all known congeners and L-*allo*-isoleucine in some of them (Fig. 1). It is an amphiphilic molecule, with the l-serine and L-*allo*-threonine residues comprising the polar part, and the remaining amino acids together with the alkyl chain making the apolar part. It penetrates the outer membrane and diffuses through the inner membrane to bind to LspA [11], sterically blocking the active site for real prolipoprotein substrates. Recently, a crystal structure of LspA from *P. aeruginosa* complexed with globomycin at 2.8 Å has been reported. It has been proposed that the Leu-Ile-Ser tripeptide in globomycin sits in the active site mimicking the “lipobox”, a four amino acid consensus sequence found in all prolipoproteins where the cleavage catalysed by LspA takes place [12].

Synthetic analogues of globomycin have been generated in structure-activity relationship studies to try to improve activity and efficacy against Gram-negative pathogens [13,14]. Somewhat surprisingly, the gene cluster encoding the biosynthesis of globomycin and congeners has not been identified so far. Here we report the identification and heterologous expression of the globomycin biosynthetic gene cluster (BGC) from the globomycin-producing *Streptomyces* sp. CA-278952, paving the way for further, in-deep studies of its biosynthesis and providing an alternative route to synthetic approaches for the generation of novel derivatives with improved properties.

## 2 Material and methods

### 2.1 Bacterial strains

The strain *Streptomyces* sp. CA-278952 was isolated from a soil sample collected at the Dílar riverbank, Granada (Spain). The heterologous hosts *Streptomyces coelicolor* M1146 and *Streptomyces albus* J1074 were kindly provided by Prof Mervyn Bibb (JIC, United Kingdom) and Prof Sergey Zotchev (University of Vienna, Austria) respectively. *Streptomyces griseofuscus* DSM40191 was obtained from the DSMZ strain collection (Braunschweig, Germany) as a freeze-dried pellet and its genome has been sequenced [15]. Its derivative *S. griseofuscus* DEL2 was created in our laboratory [16]. Strain OneShot Mach1 (Thermo Fisher Scientific; C862003) was used for the maintenance and cloning of the plasmids in this work. *E. coli* ET 12567 (pUB307), known for its non-methylation ability, was received from Y. Tong (NPGM group, DTU, Denmark) and was used for setting intergeneric conjugations with *Streptomyces* strains. BAC-optimized replicator v2.0 electrocompetent *E. coli* cells (Lucigen, Middleton, USA; 60,210–1) were used during the cloning of the globomycin BGC.

### 2.2 Culture conditions and microbial broths extraction

For genetic engineering related purposes, all *Streptomyces* strains were grown on MS solid media [17], suitable for their sporulation. For the preparation of gel blocks, *Streptomyces* sp. CA-278952 was grown in ISP2 liquid medium (International Streptomyces Project medium 2) for 24 h (28 °C, 180 rpm) until reaching the approximate end of the exponential phase. The conjugation experiments for all strains were set up according to the protocol previously described [17], omitting the spore shocking step. Exconjugant colonies were picked on MS plates supplemented with apramycin sulphate (50 ng/μL).

On the other hand, for fermentation related purposes, seed cultures of *Streptomyces* strains were typically obtained with a freshly thawed inoculum stock cultivated in ATCC-2 medium (soluble starch 20 g/L, glucose 10 g/L, NZ amine type E 5 g/L, meat extract 3 g/L, peptone 5 g/L, yeast extract 5 g/L, calcium carbonate 1 g/L, pH 7) and grown in an orbital shaker for 3 days (28 °C, 220 rpm, 70% relative humidity). *Streptomyces* seeds were then employed to inoculate 10 mL of the different fermentation media in 40 mL EPA tubes (25 × 80 mm) and grown in an orbital shaker for 7 days (28 °C, 220 rpm, 70% relative humidity). Microbial broth extracts were generated by adding 10 mL of acetone to each of the fermentation EPA tubes and shaking at 220 rpm for 1 h. Samples were centrifuged, and 12 mL of supernatant were transferred to new tubes containing 0.6 mL of DSMO. The mixtures were subjected to partial evaporation with a heated nitrogen stream to a final volume of 3 mL (20% DMSO in water).

### 2.3 LC-ESI-TOF analysis of microbial extracts

The microbial broth extracts were filtered (0.2 μm) and analysed by LC-UV-MS employing an Agilent 1100 single quadropole LC-MS system, or a Bruker maXis QTOF high-resolution mass spectrometer coupled to a HPLC system as previously described [18]. Identification of globomycin and congeners was performed using an in-house-developed application where the DAD (UV–vis) spectra, retention time, and positive and negative mass spectra of the samples are compared to the corresponding UV-LC-MS data (obtained in the same exact experimental conditions) of known microbial metabolites standards stored in a proprietary database that contains over 1200 metabolites from bacteria and actinomycetes. Analysis of globomycin and congeners production ratios was based on measurement of the areas of the integrated Extracted Ion Chromatograms (EIC) using for each of the compounds the peak generated by the monoisototopic accurate mass of the M + H adduct ± 0.005 Da.

### 2.4 DNA isolation and sequencing of *Streptomyces* sp. CA-278952 genome

Cultivation of *Streptomyces* sp. CA-278952 was done in liquid yeast extract-malt extract (YEME) medium and grown at 30 °C at 150 rpm [17]. gDNA was extracted using the Qiagen Genomic Tip 100 kit (Venlo, Netherlands) according to the instructions of the manufacturer.

For Pacbio sequencing, gDNA was shredded using a g-TUBE device (Covaris Inc., Woburn, MA, USA) and size selected using the BluePippin system (Sage Science, MA, USA). Pacbio RS II sequencing data was generated by Macrogen Inc. (Seoul, South Korea) using the 8Pac v3 kit, DNA/polymerase binding kit P6 and single-molecule real-time SMRT cell. Sub-read generation and adapter removal was performed using SMRT Analysis software (v2.3) with default software parameters yielding 106,544 reads with an N50 value of 11,671 nt. An assembly of Pacbio data was created using Flye (v2.9-b1768), applying the --pacbio_raw and --iterations 5 for five rounds of polishing [19].

Illumina MiSeq data was generated from a KAPA HYPRplus library (Roche, CH) with a size selection of approximately 450–600 nt resulting in 3,341,926 read clusters (2 × 150 nt). Illumina reads were trimmed using Trim Galore with Cutadapt (2.10) applying the switches --length 100 and --quality 20 (Marti, 2011). Illumina data was aligned to the Pacbio assembly using bowtie2-align (v2.3.4.1) with 97.22% overall alignment rate [20]. Finally, the Pacbio assembly was polished with the Illumina data using the Polypolish tool (v0.5.0) [21], resulting in 70 changes in base positions and further corrected using POLCA (v4.0.5) [22].

The topology of the genome was determined from the Flye assembly repeat graph and consisted of a single, linear chromosome, with terminal inverted repeats of 36 kb, a total assembly length of 8,211,675, a GC content of 71.5% and a mean Pacbio coverage of 81. *Streptomyces* sp*.* CA-278952 was classified by GTDB-tk (v.2.1.1) [23] as *Streptomyces* sp 000377,965 at 95.65% ANI. The BUSCO score [24] was 99.7% complete BUSCO Genes (v5.1.2) from the actinobacteria_class_odb10 lineage whereof one gene was fragmented and one existing in duplicate. Prokka (1.14.6), with extra databases as in [25], was used for annotation yielding 7095 CDSs of which 6332 were functionally annotated. The data is available under NCBI-Bioproject PRJNA883035.

### 2.5 Inactivation of the globomycin BGC in *Streptomyces* sp. CA-278952

In order to inactivate the putative globomycin BGC by introducing STOP codons to core genes via CRISPR base editing, CRISPR cBEST plasmids were constructed according to the protocol previously described [26]. All spacer sequences were selected with the help of CRISPy-web [27]. The procedure of ssDNA oligo bridging was used for the integration of spacers into plasmids. The plasmid was digested with NcoI at 37 °C for 1 h and dephosphorylated using FastAP at 37 °C for another 30 min with subsequent inactivation by incubation at 65 °C for 10 min. The 20 nt spacers were ordered as oligos from Integrated DNA Technologies (IDT, Coralville, USA) with 20 nt overlaps to the backbone. Three spacers were tested in this work: TET0001 (CGGTTGGTAGGATCGACGGCCGCAACGACCAGTCCACAAAGTTTTAGAGCTAGAAATAGA), TET0003 (CGGTTGGTAGGATCGACGGCGCAGCTGACCTACCAACAGGGTTTTAGAGCTAGAAATAGA), TET0004 (CGGTTGGTAGGATCGACGGCGCCCAGTACGTCTCCACCTGGTTTTAGAGCTAGAAATAGA) that were used in constructing plasmids pL0001, pL0003 and pL0004 respectively. The correct assembly of the plasmids was checked using Sanger sequencing employing primers TET0169 (TGTGTGGAATTGTGAGCGGATA) and TET0170 (CCCATTCAAGAACAGCAAGCAG). Correctly assembled plasmids were transferred to ET 12567 (pUB307) through electroporation. Apramycin resistant colonies of ET strain were used for intergeneric conjugation with strain CA-278952 as described above. Apramycin resistant exconjugants were used for colony PCR with the following primers: TET0023 (GCAGGCTCTCGTACCAGTAC) and TET0024 (GACGCGGAGTTCTTCGGTAT) for screening of pL0001; TET0027 (CTGTCGAGACGTCCGAGATG) and TET0028 (CCCGGTGTCCGTTATCTTCC) for screening of pL0003; and TET0030 (CAGGAACACCGCTTCGAATG) and TET0031 (TTTCATCGCCGACCCCTATG) for screening of pL0004. The PCR reaction was carried out with the Q5 High Fidelity Master Mix (New England Biolabs; M0492S), following the manufacturer's instructions. The spores for colony PCR were harvested from plates using sterile toothpicks and suspended in 50 μL of DMSO. These suspensions were first boiled and then frozen for 10 min twice. 1 μL of the suspension was then used directly for setting up PCR reactions. PCR products were Sanger sequenced using their respective primers to confirm the presence of the desired mutations. Only the introduction of pL0004 led to the generation of the desired STOP codon (W2935STOP in *globH*). Introduction of pL0001 and pL0003 led to silent single nucleotide polymorphism.

### 2.6 Cloning of the putative globomycin BGC

The globomycin BGC was cloned according to the CATCH cloning protocol [28] with the following modifications. The gel blocks, that contain *Streptomyces* sp. CA-278952 genomic DNA were prepared using 24 h grown ISP2 cultures of the strain, employing the protocol “standard procedure for preparing *Streptomyces* chromosomal DNA for PFGE” previously described [17]. Agarose blocks were stored in TE buffed at 4 °C before being subjected to digestion. The sgRNAs employed during the digestion (globo1 (protospacer sequence “GCTCGTGGAGAGATCCGACG”) and globo3 (protospacer sequence “GTACGTGGAGCGAATCCGCG”)) were designed upstream and downstream of the cloned region employing Geneious software v9.1.8 (Dotmatics, Boston, USA) and ordered as 2 nmol Alt-R CRISPR-Cas9 lyophilised and ready-to-dissolve fragments (Integrated DNA Technologies, Iowa, USA). Both sgRNAs were dissolved in RNAse-free water directly prior to use. For the primary assembly of the globomycin BGC-containing DNA fragment, pXJ157 plasmid (p15a-based plasmid with *cml*^*r*^ and *gfp* genes, I-SceI recognition site; source: X. Jiang, DTU, Denmark) was used. The overhangs were added using PCR with primers TET0129 (“CACCTACAGGGCGGGTGGATGCCGACGCGCTCGGCCGCGTCTTTAAGAAGGAGATATACCATGAGC”) and TET0130 (“CAGCCGACGAGGTGGTACGGGCCGTTCGGGTCGACCTCGCAATTGTTATCCGCTCACAATTCC”). The PCR reaction was carried out with Q5 High Fidelity Master Mix (New England Biolabs, NEB; M0492S), following the manufacturer's instructions. After amplification direct overnight digestion of PCR reactions with DpnI and subsequent gel purification using NucleoSpin Gel Clean-up kit (Macherey-Nagel, Düren, Germany) was carried out. Purified vector fragments were used for assembly with globomycin BGC-containing fragment. In-gel Cas9 digestion was performed as previously described [28], with few minor modifications: only one agarose block was used for the digestion, DTT was omitted from the digestion mixture, and Cas9 reaction buffer, supplied together with the Cas9 enzyme (NEB, M0386 M), was used instead of the cleavage buffer. Ligation and electroporation were carried out as previously described [28], except for the use of NEBuilder HiFi DNA assembly mix (NEB, E2621) instead of the Gibson mix, and the employment of BAC-optimized replicator v2.0 electrocompetent *E. coli* cells (Lucigen, Middleton, USA). At least 10 white, chloramphenicol resistant colonies were used for plasmid isolation and screening by Nanopore sequencing (Nanopore sequencing description below). Positive plasmids that contained the globomycin BGC were used for the second round of construction, namely adding the *apr*-*attP*-*int* fragment for apramycin resistance, necessary for the integration of the final construct into the *Streptomyces* chromosome. Fragment *apr*-*attp*-*int* was amplified using primers Xj483 (“GAGTGGCAGGGCGGGGCGTAATAGGGATAAGGTTCATGTGCAGCTCCATCAGCAA”), Xj484 (“GGATGATTAATTGTAAAATTACCCTGTTATGGAAACCTGTCGTGCCAGCCTGC”) and plasmid pRM4 as a template (source: X. Jiang, DTU, Denmark). PCR reactions were set-up using Q5 High Fidelity Master Mix (NEB; M0492S) according to the manufacturer's instructions. The fragment of 4.2 Kbp size was excised from the gel and purified using NucleoSpin Gel Clean-up kit (Macherey-Nagel) and used for the assembly with I-SceI-digested globomycin BGC-containing plasmids using NEBuilder HiFi DNA assembly mix (New England Biolabs, Ipswich, USA). Apramycin- and chloramphenicol-resistant colonies were used for pGlobo plasmid isolation and Nanopore sequencing, as described below, to confirm the presence of the complete globomycin BGC fragment. *E. coli* ET 12567 (pUB307) cells were electroporated with correctly assembled pGlobo plasmids and subsequently used for their transfer to heterologous hosts through the process of intergeneric *E. coli*-*Streptomyces* conjugation (as described above). Apramycin resistant colonies of exconjugant heterologous hosts were used for PCR-based verification of the presence of the globomycin BGC, using primers binding upstream (TET0033 (CAAGGGAGACATGCGGTGAT) and TET0034 (CGTGGACATCAGGGTGAACA)), center (TET0047 (GGGTGTCCGTGGTTCGTTC) and TET0048 (GGAGGATTTGTGGGAGTTGGT)), and downstream (TET0037 (TAGAACTGACGTGCCGACAC) and TET0038 (CATCTCGGACGTCTCGACAG)) of the cloned region. The spore suspensions and PCR set up were carried out as described above. Triple positive exconjugant colonies were selected for carrying out the fermentation and verification of globomycin production.

### 2.7 DNA isolation and sequencing of pGlobo

The BGC containing version of the vector and final pGlobo construct were sequenced using in-house Nanopore runs as described below to confirm presence of full, uninterrupted globomycin BGC-containing fragment. Cultivation of Mach1 cells prior to extraction, carrying the pXJ157 containing the globomycin BGC or pGlobo, was done in LB with 50 μg/mL of apramycin growing overnight at 200 rpm and 37 °C and used for the inoculation of 500 mL fresh LB supplemented with 50 μg/mL of apramycin in a 2 L baffled shake flask the following day. The culture was incubated at 200 rpm at 37 °C overnight followed by isolation of the plasmid by using the NucleoBond ® BAC 100 Kit (Macherey-Nagel, Germany) according to the manufacturer's instructions. A library was constructed using the Rapid Barcoding kit (SQK-RBK004, Oxford Nanopore technologies, Inc.) and run on a FLO-MIN106D (R9.4.1) flow cell on a Nanopore MinION device. Base calling and demultiplexing was done using Guppy (v5.0.11+2b6dbff) in high accuracy (hac) mode. The fastq files were used for creating an alignment with the genome of *Streptomyces* sp. CA-278952 using minimap2 [29] in order to confirm accuracy of the construct.

## 3 Results and discussion

### 3.1 Identification and bioinformatic analysis of the globomycin BGC

During our ongoing efforts towards the discovery of new Gram-negative active natural products we identified *Streptomyces* sp. CA-278952 as a producer of globomycin and congeners. The genome of the strain was sequenced employing a combination of PacBio (Macrogen Inc. South Korea) and Illumina to assemble in a single 8.2 Mbp contig with a 71.5% GC content. antiSMASH analysis [30] predicted 33 regions potentially encoding the biosynthesis of secondary metabolites.

**Fig. 2:**
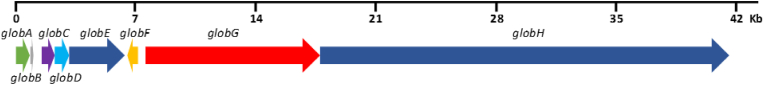
Putative globomycin biosynthetic gene cluster.

A retro biosynthetic analysis of globomycin allowed us to identify a hybrid PKS-NRPS cluster as the most likely candidate for globomycin biosynthesis (Fig. 2, SI Table 1) [31]. There are three type I polyketide synthase (PKS) modules, two in GlobG and one in GlobH, which are proposed to synthesise the alkyl chain which varies in length depending on the congener. Whether this is the consequence of iterative PKS action, recruitment of different starting/elongation units or combination of both remains an open question. The ketosynthase domain from module 1 contains the initiation factor KSQ, with the highly conserved glutamine residue required for decarboxylation of the first methylmalonyl-CoA unit [32]. Alignment of the amino acid sequences of the AT domains allowed us to identify canonical signatures for malonyl-CoA (module 2) and methylmalonyl-CoA (modules 1 and 3) selection. Additionally, the KR domains from modules 2 and 3 belong to the B1 subtype [33]. Finally, the two dehydratase domains found in modules 2 and 3 contain the canonical catalytic histidine and aspartic residues [34]. The dehydratase domain at module 3 must be inactive, or a domain-skipping process must take place, since the hydroxyl group generated by the ketoreductase is essential for the cyclization of the depsipeptide.

**Fig. 3:**
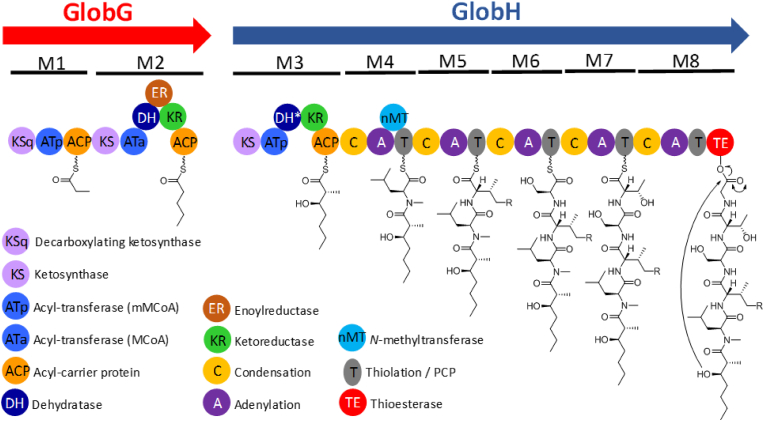
Proposed biosynthetic pathway of globomycin and congeners. The figure depicts biosynthesis of SF-1902 A_2_ (where R = CH_3_ and n = 1 in Fig. 1). The asterisk denotes proposed inactive domains.

The antiSMASH adenylation domain substrate specificity analysis of the five non-ribosomal peptide synthetase (NRPS) modules found in GlobH predicted a peptide backbone comprising Val (Leu required), Val (Val or *allo*-Ile required), Ser (Ser required), undetermined (*allo*-Thr required) and Gly (Gly required) for modules 4 to 8, respectively, and therefore is in fairly good agreement with the chemical structure of globomycins. Relaxed substrate specificity of the adenylation domain at module 5 would allow selection of both L-*allo*-isoleucine and valine, thus accounting for the biosynthesis of different globomycin congeners. As expected, module 4 contains a *N*-methyltransferase domain, responsible for the N-methylation of leucine. Taking these predictions together, and assuming fulfilment of the collinearity rule, we can propose the biosynthesis outlined in Fig. 3.

Globomycin and all known congeners contain the non-proteogenic amino acid L-*allo*-threonine, incorporated into the backbone by module 7. Additionally, globomycin and congeners SF-1902 A_5_, SF-1902 A_4a_ and SF-1902 A_2_ contain the non-proteogenic amino acid L-*allo*-isoleucine, incorporated by module 5. A tblastn search in the genome of *Streptomyces* sp. CA-278952 allowed us to identify, around 20 kbp upstream of *globG*, two adjacent genes encoding for proteins homologous to DsaD (53% identity, 64% similarity) and DsaE (52% identity, 63% similarity), which have been biochemically characterized and shown to generate L-*allo*-isoleucine from l-isoleucine [35].

On the other hand, and to the best of our knowledge, the bacterial biosynthetic origin of L-*allo*-threonine remains to be elucidated. Interestingly, *globE* encodes for a NRPS adenylation-thiolation didomain protein. Analysis of the substrate specificity of the adenylation domain suggests preference towards threonine. The adjacent gene *globD* encodes for a NAD(P)H-dependent oxidoreductase of the SDR family. A tblastn search of adjacent GlobD and GlobE homologous proteins in the non-redundant nucleotide NCBI database allowed us to identify the pairs HmtG (70% identity, 81% similarity to GlobD)/HmtF (49% identity, 58% similarity to GlobE) from the himastatin BGC in *Streptomyces himastaticus* ATCC 53653 [36]; and WS21 (68% identity, 81% similarity to GlobD)/WS22 (38% identity, 48% similarity to GlobE) from the WS9326A BGC in *Streptomyces* sp. SNM55 [37]. Himastatin is a dimeric cyclohexadepsipeptide antibiotic that contains d-threonine in its structure, presumably derived from the epimerization of the α carbon of L-*allo*-threonine catalysed by the epimerization domain found in the third module of the NRPS protein HtmL [36]. On the other hand, WS9326A is a cyclodepsipeptide that contains a L-*allo*-threonine in its structure, which has been proposed to be shuttled to the nascent non-ribosomal peptide *in trans* by WS22 and the type II TE protein WS20 [37]. The specificity of the adenylation domain of WS22 towards l-threonine has been experimentally confirmed, but no data on its epimer L-*allo*-threonine is available [38]. Taken together, these data prompt us to propose that GlobD/GlobE in the globomycin BGC, as well as HmtG/HmtF in the himastatin BGC and WS21/WS22 in the WS9326A BGC, are involved in the biosynthesis of L-*allo*-threonine from l-threonine. We hypothesise that l-threonine is selected and loaded into the thiolation domain of GlobE/HmtF/WS22, followed by oxidation of the β-hydroxy group and subsequent stereoselective reduction of the generated β-keto group (with overall inversion of the β carbon configuration) catalysed by GlobD/HmtG/WS21 to biosynthesize L-*allo*-threonine (SI, Figure S1). Interestingly, in the case of himastatin and WS9326A, L-*allo*-threonine is then presumably introduced in *trans* via NRPS modules lacking an adenylation domain (third module of HmtL for himastatin, first module of WS19 for WS9326A) in a process mediated by a type II thioesterase [37]. However, in the case of globomycin and congeners, all the NRPS modules follow a canonical arrangement and contain adenylation domains. Nonetheless, the type II thioesterase encoded by *globC* in the globomycin BGC could be involved in the shuttle of L-*allo*-threonine through module 7 in a similar way to that reported for WS9326A [37]. Alternatively, GlobC might catalyse the hydrolysis of the thioester linkage to release L-*allo*-threonine, followed by selection and loading of the free L-*allo*-threonine by the adenylation domain at module 7.

Finally, *globF* encodes for a signal peptidase II (LspA) protein. A second LspA homolog is encoded elsewhere in the genome of strain CA-278952. *Myxococcus xanthus* DK1622, the producer of myxovirescin, contains four copies of *lspA* in the genome, with two of those copies, *lspA3* and *lspA4*, located in the myxovirescin BGC. It has been proposed that LspA3 and LspA4 could function as SPAseIIs and also play a role in the myxovirescin resistance and biosynthesis regulation [39]. Therefore, it seems reasonable to hypothesise that GlobF might play a role in globomycin resistance and/or biosynthesis regulation in *Streptomyces* sp. CA-278952. Somewhat surprisingly, a protein alignment of GlobF and the second LspA copy in the genome of *Streptomyces* sp. CA-278952 shows no differences in the 14 amino acid residues identified as strictly conserved among 485 LspA from different bacterial species [12].

### 3.2 Inactivation of the globomycin BGC in *Streptomyces* sp. CA-278952

**Fig. 4:**
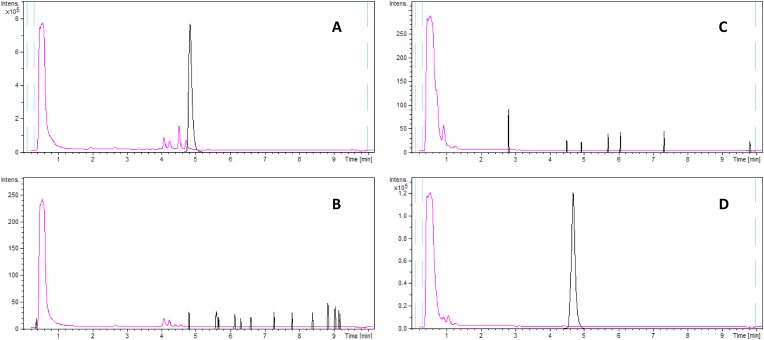
LC-ESI-TOF HRMS analysis of *Streptomyces* sp. CA-278952 knock-out mutant and globomycin heterologous expression in *S. albus* J1074. UV profile at 210 nm (purple trace) and Extracted Ion Chromatogram at 656.4229 ± 0.005 (black trace, C_32_H_58_N_5_O_9_^+^, [M+H]^+^ adduct of globomycin) from the microbial broth extracts of *Streptomyces* sp. CA-278952 wild type (FRM medium) (**A**); *Streptomyces* sp. CA-278952 *globH* knock-out mutant (FRM medium) (**B**); *Streptomyces albus* J1074 pXJ157 negative control (empty vector) (NOC-2 medium) (**C**); *Streptomyces albus* J1074 pGlobo (carrying globomycin BGC) (NOC-2 medium) (**D**).

Once a candidate BGC for globomycin and congeners biosynthesis was identified, a null mutant was created employing CRISPR-cBEST [26]. A W2935STOP replacement in the PKS-NRPS gene *globH* was carried out, thus generating a truncated, non-functional GlobH protein. The null mutant was then subjected, together with the wild type CA-278952 as a positive control, to an exhaustive fermentation study employing 16 different fermentation media, and the microbial broth extracts were analysed by LC-HRMS. Neither globomycin nor any of its congeners could be detected in any of the null mutant broth extracts, in contrast to that of the wild type strain, where globomycin and derivatives were detected in significant amounts in most of the fermentation conditions (Fig. 4, SI Figure S2). These data strongly suggested the involvement of GlobH in the biosynthesis of globomycin and congeners.

### 3.3 Heterologous expression of the globomycin BGC

To further confirm the identity of the globomycin and congeners BGC, a genomic region spanning approximately 51.1 kbp containing the putative biosynthetic pathway, but lacking the DsaD and DsaE homologues proposed to biosynthesize L-*allo*-isoleucine, was cloned into the vector pXJ157 using a modified CATCH procedure [28] and transferred via biparental conjugation to four different *Streptomyces* sp. heterologous hosts: *S. albus* J1074, *S. coelicolor* M1146, *S. griseofuscus* wt, and *S. griseofuscus* DEL2 (a genome-minimized mutant of *S. griseofuscus* wt). The globomycin-BGC carrying strains, their corresponding negative controls (each of the heterologous hosts with the integrated empty pXJ157 vector) and *Streptomyces* sp. CA-278952 wt as positive control were grown in 8 different fermentation media, selected as the best conditions for globomycin and congeners production in the wild type strain, and the microbial broth extracts were analysed by LC-HRMS. Globomycin and congeners were heterologously produced in *S. albus* J1074 and to a much lesser extent in *S. coelicolor* M1146 (Fig. 4, SI Figure S3). No production could be detected in any of the remaining heterologous hosts, which underscores the importance of employing different heterologous hosts, even if they belong to the same genus. Interestingly, qualitative analysis of the relative globomycin congeners production ratios in *S. albus* J1074 employing the areas of their corresponding peaks in the Extracted Ion Chromatograms indicated that the major component is SF-1902 A_3_, in contrast to the wild type strain, where this is a minor congener and the major component in all the fermentation conditions is globomycin. SF-1902 A_3_ contains a valine residue in place of the L-*allo*-isoleucine found in globomycin. Bioinformatic analysis of the genome of *S. albus* J1074 shows that it lacks a DsaE homolog, and therefore production of L-*allo*-isoleucine is likely to be impaired. Thus, biosynthesis of globomycin congeners lacking this amino acid, such as SF-1902 A_3_, is enhanced.

## 4 Conclusions

Globomycin (and congeners) is one of the only two microbial natural products known to block the action of the signal peptidase LspA, a key protein in the maturation and secretion pathway of lipoproteins. LspA is essential in Gram-negative bacteria and has recently attracted much attention as a possible target to develop new antibiotics against Gram-negative pathogens. We have employed a genome-mining strategy in a globomycin-producing *Streptomyces* strain to identify its putative biosynthetic gene cluster. CRISPR-base editing was employed to target one key PKS-NRPS gene from the BGC and generate a null mutant, which was completely unable to produce globomycin and congeners. Furthermore, the BGC was cloned and heterologously expressed in *S. albus* J1074 and *S. coelicolor* M1146, thus unambiguously demonstrating that it is indeed responsible of globomycin and congeners biosynthesis. Our work paves the way for further studies on the biosynthesis of this family of cyclic lipodepsipeptides, including the biosynthetic origin of L-*allo*-threonine and the mechanism by which the different-length alkyl chains are generated. It also provides an alternative to synthetic methodologies for the generation of novel globomycin derivatives with improved pharmacological properties.

## CRediT authorship contribution statement

**Daniel Oves-Costales:** Formal analysis, Investigation, Conceptualization, Writing - original draft, Writing - review & editing. **Tetiana Gren:** Conceptualization, Formal analysis, Investigation, Writing – original draft, Writing – review & editing. **Eva Baggesgaard Sterndorff:** Conceptualization, Investigation, Writing – original draft, Writing – review & editing. **Jesús Martín:** Investigation, Software. **Francisco Javier Ortiz-López:** Investigation, Writing – review & editing. **Tue S. Jørgensen:** Investigation, Data curation, Formal analysis. **Xinglin Jiang:** Investigation. **Fernando Román-Hurtado:** Investigation, Writing – review & editing. **Fernando Reyes:** Supervision, Writing – review & editing. **Olga Genilloud:** Conceptualization, Supervision, Project administration, Funding acquisition, Writing – review & editing. **Tilmann Weber:** Conceptualization, Supervision, Project administration, Funding acquisition, Writing – review & editing.

## Declaration of competing interest

There are no conflicts of interest to declare.
