# Supplementary material for: Identification and heterologous expression of the globomycin biosynthetic gene cluster
Source: Synth Syst Biotechnol. 2023 Feb 7;8(2):206–12. doi: 10.1016/j.synbio.2023.02.001 (PMC9943842; doi:10.1016/j.synbio.2023.02.001)
Supplement: Multimedia component 1 [file mmc1.docx]

**Supplementary Information**

**Identification and heterologous expression of the globomycin biosynthetic gene cluster**

Daniel Oves-Costales^1§^, Tetiana Gren^2§^, Eva Baggesgaard Sterndorff^2^, Jesús Martín^1^, Francisco Javier Ortiz-López^1^, Tue S. Jørgensen^2^, Xinglin Jiang^2^, Fernando Román-Hurtado^1^, Fernando Reyes^1^, Olga Genilloud^1^, Tilmann Weber^2^

^1^Fundacion MEDINA, Centro de Excelencia en Investigación de Medicamentos Innovadores en Andalucía, Avda del Conocimiento 34, 18016 Armilla, Granada, Spain

^2^The Novo Nordisk Foundation Center for Biosustainability, Technical University of Denmark, Kemitorvet, building 220, 200 Kgs. Lyngby, Denmark

^§^These authors contributed equally to this work.

Correspondence should be addressed to: Daniel Oves-Costales at [daniel.oves@medinaandalucia.es](mailto:daniel.oves@medinaandalucia.es), Tilmann Weber at [tiwe@biosustain.dtu.dk](mailto:tiwe@biosustain.dtu.dk) and Olga Genilloud at [olga.genilloud@medinaandalucia.es](mailto:olga.genilloud@medinaandalucia.es)

**Table of Contents**

Composition of *Streptomyces* sp. fermentation media

**Table S1**. Putative functions of proteins encoded in the *glob* biosynthetic gene cluster

**Figure S1**. Proposed biosynthetic pathway of L-*allo*-threonine from L-threonine.

**Figure S2**. LC-ESI-TOF HRMS analysis of globomycin and congeners production in *Streptomyces* sp. CA-278952 wild type and *Streptomyces* sp. CA-278952 *globH* knock-out mutant.

**Figure S3**. LC-ESI-TOF HRMS analysis of globomycin and congeners production in *Streptomyces albus* J1074 pXJ157-globo-int-apr, *Streptomyces albus* J1074 pXJ157 and *Streptomyces* sp. CA-278952 wt.

**Composition of *Streptomyces* sp. fermentation media**

Fermentation medium CLA: yeast autolysate (5 g/L), corn meal (40 g/L), lactose (40 g/L), pH adjusted to 7.0

Fermentation medium DEF-15S: magnesium chloride hexahydrate (1 g/L), ammonium chloride (2 g/L), calcium carbonate (2 g/L), dipotassium hydrogen phosphate (1 g/L), sodium chloride (1 g/L), sucrose (5 g/L), sodium sulphate (2 g/L), soluble starch from potato (20 g/L), trace elements mix (1 mL/L; composition per 100 mL: MnCl_2_*4H_2_O 100 mg, ZnCl_2_ 100 mg, FeCl_2_*4H_2_O 100 mg, NaI 50 mg).

Fermentation medium DNPM: Dextrin from corn type I (40 g/L), MOPS (21 g/L), N-Z Soy BL (7.5 g/L), yeast extract (5 g/L), pH adjusted to 7.0

Fermentation medium FPY-12: amicase (5 g/L), bacto peptone (5 g/L), fructose (20 g/L), glucose (10 g/L), maltose (10 g/L), trace elements mix (1 mL/L; composition per 100 mL: FeSO_4_*7H_2_O 500 mg, ZnSO_4_*7H_2_O 500 mg, MnSO_4_*H_2_O 100 mg, CuSO_4_*5H_2_O 50 mg, CoCl_2_*6H_2_O 50 mg), pH adjusted to 7.0

Fermentation medium FPY-6: amicase (5 g/L), bacto peptone (2 g/L), fructose (20 g/L), NZ amine E (5 g/L), yeast extract (5 g/L), trace elements mix (1 mL/L; composition per 100 mL: FeSO_4_*7H_2_O 500 mg, ZnSO_4_*7H_2_O 500 mg, MnSO_4_*H_2_O 100 mg, CuSO_4_*5H_2_O 50 mg, CoCl_2_*6H_2_O 50 mg), pH adjusted to 7.0

Fermentation medium FR23: cane molasses (20 g/L), glucose (5 g/L), cottonseed flour (20 g/L), soluble starch from potato (30 g/L), pH adjusted to 7.0.

Fermentation medium FRM: bacto soytone (3 g/L), calcium carbonate (3 g/L), dextrin from corn type I (20 g/L), glycerol (20 g/L), ammonium sulphate (2 g/L), pH adjusted to 7.4.

Fermentation medium KHC: beta cyclodextrin (10 g/L), CoCl_2_*6H_2_O (5 mg/L), dextrin from corn type I (20 g/L), yeast extract (10 g/L), tomato paste (20 g/L)

Fermentation medium MPG: glucose (10 g/L), millet meal (20 g/L), MOPS (20 g/L), cottonseed flour (20 g/L), pH adjusted to 7.0.

Fermentation medium M016: glucose (10 g/L), soluble starch from potato (10 g/L), maltose (10 g/L), yeast extract (1 g/L), soytone peptone (5 g/L), tryptone (4 g/L), KH_2_PO_4_ (0.1 g/L), K_2_HPO_4_, (0.2 g/L), MgSO_4_.7H2O (0.05 g/L), NaCl (0.02 g/L), CaCl_2_*2H_2_O (0.05 g/L), trace elements mix (1 mL/L; composition per 1000 mL: SnCl_2_ 0.005 g, H_3_BO_3_ 0.01 g, NaMoO_4_ 0.012 g, CuSO_4_ 0.015 g, CoCl_2_ 0.02 g, KCl 0.02 g, ZnCl_2_ 0.02 g, MnSO_4_ 0.1 g, FeCl_3_ 5.8 g).

Fermentation medium NOC-2: bacto casitone (5 g/L), yeast extract (5 g/L), glycerol (20 g/L), proteose peptone (5 g/L), pH adjusted to 7.0.

Fermentation medium S9702W MOD: Calcium carbonate (5 g/L), Na_2_HPO_4_ (0.1 g/L), MgSO_4_*7H_2_0 (0.5 g/L), peptone vegetable (2 g/L), sodium pyruvate (1 g/L), soluble starch from potato (10 g/L), HEPES buffer 1 M pH 7 (3 mL), pH adjusted to 7.0.

Fermentation medium TP: calcium carbonate (4 g/L), cottonseed flour (6 g/L), sucrose (27 g/L), ammonium sulphate (4 g/L), ZnSO_4_*H_2_O (30 mg/L).

Fermentation medium M016 agar: The same than MO16 above, supplemented with 2 g/L agar and dispensed on Petri dishes after autoclaved.

Fermentation medium DNPM agar: The same than DNPM above, supplemented with 2 g/L agar and dispensed on Petri dishes after autoclaved.

Fermentation medium FR23 agar: The same than FR23 above, supplemented with 2 g/L agar and dispensed on Petri dishes after autoclaved.

**Table S1**. Putative functions of proteins encoded in the *glob* biosynthetic gene cluster.

**Figure S1**. Proposed biosynthetic pathway of L-*allo*-threonine from L-threonine.

**Figure S2**. LC-ESI-TOF HRMS analysis of globomycin and congeners production in *Streptomyces* sp. CA-278952 wild type and *Streptomyces* sp. CA-278952 *globH* knock-out mutant

Representation of the Extracted Ion Chromatogram (EIC) areas for globomycin and congeners in each of the 16 media employed in the fermentation of *Streptomyces* sp. CA-278952 *globH* knock-out mutant (left) and *Streptomyces* sp. CA-278952 wt (right). The Extracted Ion Chromatograms were obtained with the M+H^+^ adducts for each of the species: EIC at 628.3916 ± 0.005, C_30_H_54_N_5_O_9_^+^, Antibiotic SF-1902 A_2_; EIC at 670.4386 ± 0.005, C_33_H_60_N_5_O_9_^+^, Antibiotics SF-1902 A_4a_-_4b_; EIC at 642.4073 ± 0.005, C_31_H_56_N_5_O_9_^+^, Antibiotic SF-1902 A_3_; EIC at 656.4229 ± 0.005, C_32_H_58_N_5_O_9_^+^, globomycin; EIC at 684.4542 ± 0.005, C_34_H_62_N_5_O_9_^+^, Antibiotic SF-1902 A_5._

**Figure S3.** LC-ESI-TOF HRMS analysis of globomycin and congeners production in *Streptomyces albus* J1074 pGlobo, *Streptomyces albus* J1074 pXJ157 and *Streptomyces* sp. CA-278952 wt.

Representation of the Extracted Ion Chromatogram (EIC) areas for globomycin and congeners in each of the 8 media employed in the fermentation of *Streptomyces albus* J1074 pGlobo (carrying the globomycin BGC) (left), *Streptomyces albus* J1074 pXJ157 (empty vector, negative control) (center) and *Streptomyces* sp. CA-278952 wt (positive control) (right). The Extracted Ion Chromatograms were obtained with the M+H^+^ adducts for each of the species: EIC at 628.3916 ± 0.005, C_30_H_54_N_5_O_9_^+^, Antibiotic SF-1902 A_2_; EIC at 670.4386 ± 0.005, C_33_H_60_N_5_O_9_^+^, Antibiotics SF-1902 A_4a_-_4b_; EIC at 642.4073 ± 0.005, C_31_H_56_N_5_O_9_^+^, Antibiotic SF-1902 A_3_; EIC at 656.4229 ± 0.005, C_32_H_58_N_5_O_9_^+^, globomycin; EIC at 684.4542 ± 0.005, C_34_H_62_N_5_O_9_^+^, Antibiotic SF-1902 A_5._
